# ENDOCRINOLOGY IN THE TIME OF COVID-19: Management of hyperthyroidism and hypothyroidism

**DOI:** 10.1530/EJE-20-0445

**Published:** 2020-05-19

**Authors:** Kristien Boelaert, W Edward Visser, Peter Nicholas Taylor, Carla Moran, Juliane Léger, Luca Persani

**Affiliations:** 1 Institute of Applied Health Research, College of Medical and Dental Sciences, University of Birmingham, Birmingham, UK; 2 Academic Centre for Thyroid Diseases, Department of Internal Medicine, Erasmus Medical Centre, Rotterdam, The Netherlands; 3 Thyroid Research Group School of Medicine Cardiff University, University Hospital of Wales, Heath Park, Cardiff, UK; 4 Beacon Hospital, Sandyford, Dublin, Ireland; 5 Pediatric Endocrinology Diabetology Department, Reference Center for Growth and Development Endocrine Diseases, Université de Paris, Hopital Robert Debre, Paris, France; 6 Department of Clinical Sciences and Community Health, University of Milan, Milan, Italy; 7 Postgraduate School of Endocrinology and Metabolic Diseases, University of Milan, Milan, Italy; 8 Department of Endocrine and Metabolic Diseases, IRCCS Istituto Auxologico Italiano, San Luca Hospital, Milan, Italy

## Abstract

This manuscript provides guidance on the management of thyroid dysfunction during the COVID-19 pandemic. Autoimmune thyroid diseases are not linked to increased risks of COVID-19. Uncontrolled thyrotoxicosis may result in more severe complications from SARS-CoV-2 infection, including thyroid storm. The management of patients with a new diagnosis of hyperthyroidism is best undertaken with a block-and-replace regimen due to limited biochemical testing availability. Antithyroid drug (ATD)-induced neutropenia may favour the progression of COVID-19 and symptoms of infection may be confused with SARS-CoV-2 infection. The withdrawal of ATDs and urgent measurement of neutrophils should be considered in case of flu-like manifestations occurring in the initial months of treatment. Urgent surgery or 131-I may be undertaken in selected cases of uncontrolled thyrotoxicosis. Patients with COVID-19 infection may present with conjunctivitis, which could cause diagnostic difficulties in patients with new or existing Graves’ ophthalmopathy. Patients who are on replacement treatment with thyroid hormones should ensure they have sufficient supply of medication. The usual advice to increase dosage of levothyroxine during pregnancy should be adhered to. Many newly presenting and previously diagnosed patients with thyroid dysfunction can be managed through virtual telephone or video clinics supported by a dedicated nurse-led service, depending on available facilities.

## Introduction

On March 11, 2020, the World Health Organisation (WHO) declared infection by the corona (SARS-CoV-2) virus causing COVID-19 disease a global pandemic (1). Most countries have implemented local and national crisis management plans to try and cope with the most severe and overwhelming health crisis in a century. Many healthcare practitioners have been re-deployed to frontline services and other areas which are most in need and routine medical care and elective procedures have been postponed. Additionally, with more than a third of the world population in lockdown, patients face difficulties accessing facilities for clinic review as well as diagnostic and therapeutic procedures.

Hypo- and hyper-thyroidism are chronic conditions which are usually treated in an outpatient setting and their management is heavily reliant on biochemical testing, imaging and nuclear medicine procedures. Moreover, some of the commonly used therapies may pose diagnostic and therapeutic challenges for healthcare practitioners and patients. This manuscript aims to provide consensus advice for safe management of patients during a resource-limited crisis and may need to be adapted to local practice and available infrastructure.

The authors screened the available literature and included search terms like ‘thyroid and COVID-19/SARS/Corona’, ‘thyroid and influence’ and ‘thyroid and ACE-receptor’. Since no obvious evidence was found on how to handle patients with thyroid dysfunction during the COVID-19 pandemic, the authors decided to join efforts for an expert-based recommendation. Our key messages are summarised in Table 1.

**Table 1:** Thyroid dysfunction and COVID-19: key messages.

• Uncontrolled thyrotoxicosis may be associated with more severe complications from COVID-19.
• Symptoms of infection from COVID-19 are indistinguishable from those of antithyroid drug-induced neutropenia. The withdrawal of ATDs and urgent neutrophil measurement is advised.
• Block-and-replace regimens are preferred for newly diagnosed hyperthyroidism in adults and children.
• Conjunctival involvement of COVID-19 may make diagnosis of thyroid eye disease difficult and may present a particular risk of infection.
• Progression of thyroid eye disease should be prevented and advice to stop smoking and/or selenium supplementation should be reinforced.
• No particular concerns are evident for the management of patients with hypothyroidism during this pandemic.
• During pregnancy, levothyroxine dosage should be increased even if monitoring of thyroid function testing becomes difficult.
• Virtual telephone or video clinics for thyroid patients should be implemented to reduce the risks connected to hospital access.

## How will COVID-19 and thyroid dysfunction impact each other?

- *Effect of thyroid autoimmunity on COVID-19 infection*: Both hyper- and hypothyroidism are usually caused by autoimmune conditions. Around 80% of the pathogenesis of Graves’ disease and Hashimoto thyroiditis is determined by genetic factors with environmental factors accounting for 20%. Viral infections including those with parvo-, Epstein Barr and Hepatitis C viruses have been proposed as potential environmental triggers (2), but there is no evidence that patients with existing autoimmune thyroid disease are more susceptible to contracting viral illnesses including infection with SARS-CoV-2 or that they are at risk of developing more severe COVID-19 disease. Certain subsets of patients, such as those with Graves’ ophthalmopathy who are actively undergoing immunosuppressive therapy, are likely to be at increased risk of developing severe corona virus infection.
- *Effect of antithyroid drugs (ATDs) on COVID-19*: ATDs are not known to increase the risk of infection, and we do not consider patients on ATDs to be at a higher risk of contracting COVID-19 or of developing more severe disease in the event of contracting the infection.
- *Effects of COVID-19 on hypothalamic-pituitary-thyroid axis*: Systemic diseases are known to be associated with low-T3 syndrome or non-thyroidal-illness (3). Severe COVID-19 is expected to generate such condition especially when the infection is associated with fever and lower respiratory tract involvement. Additionally, SARS-CoV-2 infection has been reported to affect the nervous system, with involvement of cranial nerves for olfaction and taste commonly affected (4). Future studies are required to evaluate the risk of hypothalamitis, potentially leading to central hypothyroidism in COVID-19 patients after remission (5, 6). Nonetheless, we advise against routine screening for thyroid dysfunction in acutely ill patients unless there is a strong suspicion that thyroid disease is contributing to the clinical presentation.
- *Effect of thyroid function control on COVID-19*: There is no evidence that those with poorly controlled thyroid disease are more likely to contract viral infections. However, it is plausible that patients with uncontrolled thyroid dysfunction, especially those with thyrotoxicosis, may be at higher risk of complications (e.g. thyroid storm) from any infection (7). We strongly recommend that patients with thyroid dysfunction continue taking their thyroid medication(s) to reduce this risk. Emergency thyroid surgery or 131-I administration may be considered in life-threatening cases of uncontrolled hyperthyroidism, although the administration of radioiodine has been suspended in many countries.
- *Effect of 131-I treatment and thyroid surgery on COVID-19*: There is no evidence that patients who have received radioiodine or have had previous thyroid surgery are at increased risk of developing infection with any virus including the SARS-CoV-2 virus.

## How should patients with thyroid dysfunction be managed in a crisis setting with limited resources?

### Hyperthyroidism

**Figure 1:**
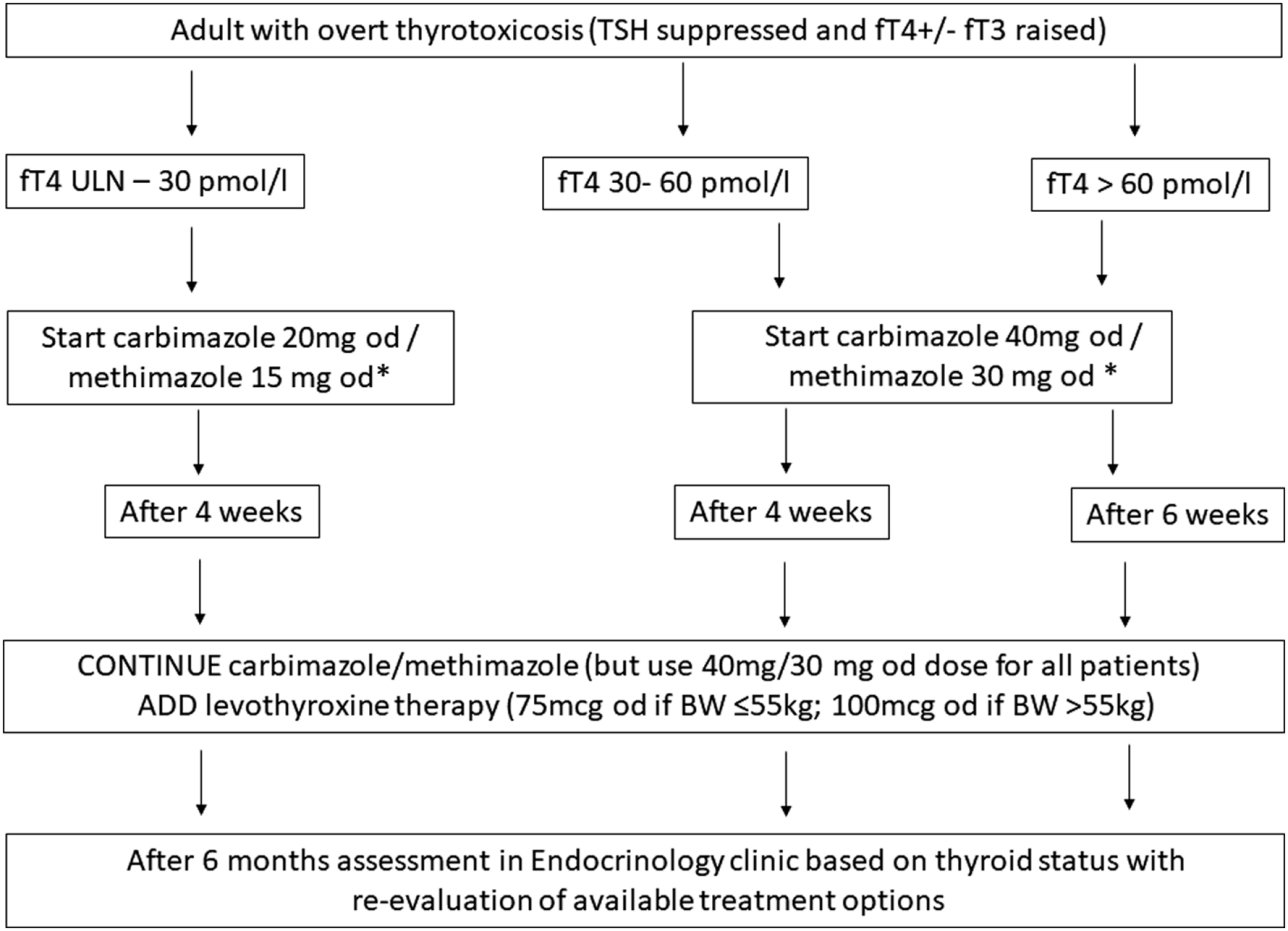
Block-and-replace algorithm for treatment of thyrotoxicosis in adults in a crisis setting with limited access to medical resources. ULN, upper limit of normal; BW, body weight. *If carbimazole/methimazole is commenced, the patient should be informed regarding potential side effects, including birth defects, agranulocytosis and abnormal liver function. Guidance on how to advise and manage patients with suspected agranulocytosis during COVID-19 is within the manuscript.

**Figure 2:**
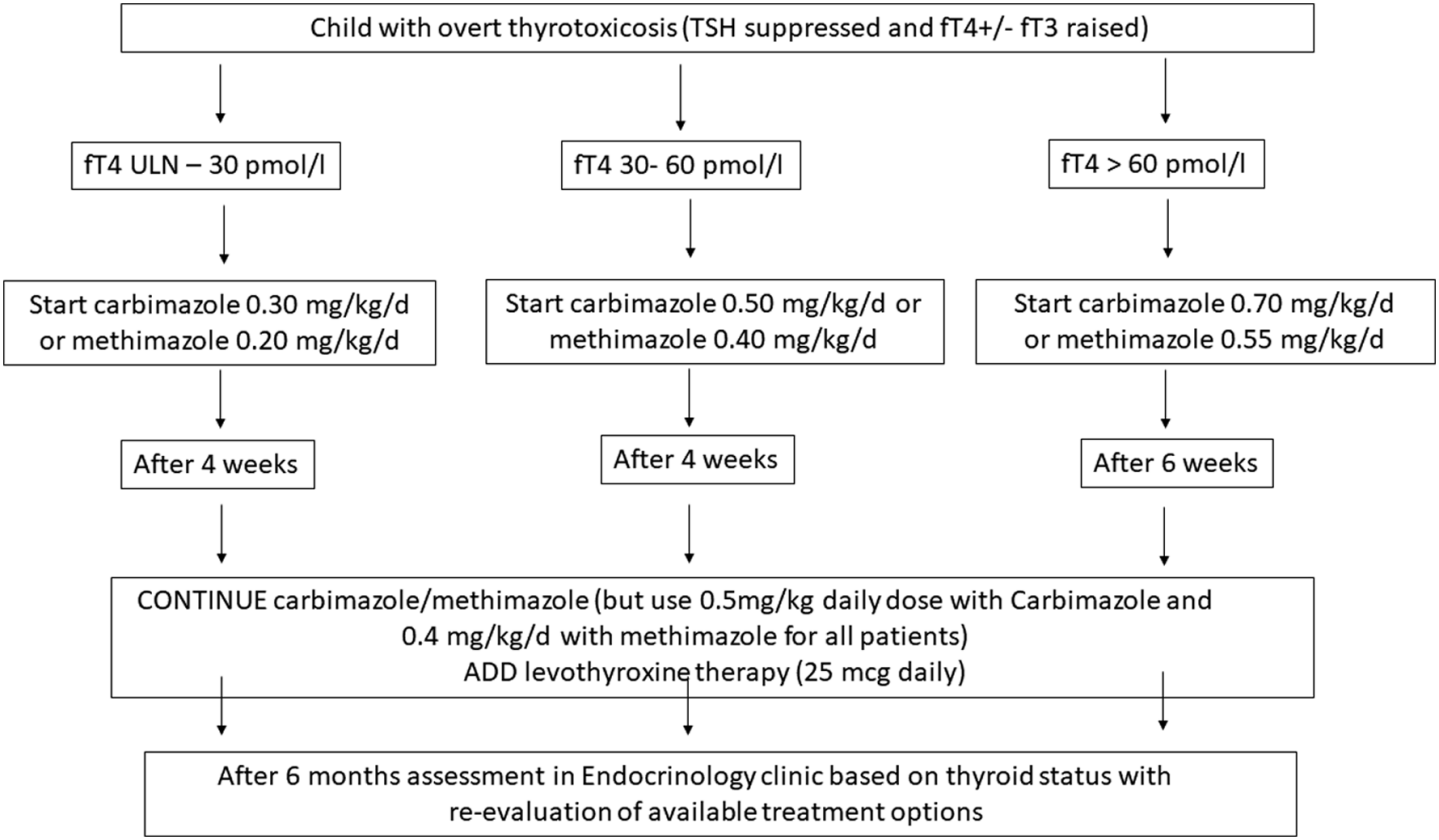
Block-and-replace algorithm for treatment of thyrotoxicosis in children in a crisis setting with limited access to medical resources. ULN, upper limit of normal; BW, body weight. *If carbimazole/methimazole is commenced, the patient should be informed regarding potential side effects, including agranulocytosis and abnormal liver function. Guidance on how to advise and manage patients with suspected agranulocytosis during COVID-19 is contained within the manuscript.
- *Differentiating between infection caused by agranulocytosis and COVID-19*: Patients taking ATDs are at risk of developing neutropenia (neutrophil count of <1.0 × 10^9^/L) or agranulocytosis (neutrophil count of <0.5 × 10^9^/L), which occurs in 0.2–0.5% of patients taking these medications, especially during the first months of treatment (7, 11). Symptoms of neutropenia (sore throat, mouth ulceration, fever and flu-like illness) may overlap with symptoms of COVID-19 (fever, new continuous cough and flu-like illness). It is difficult, if not impossible, for patients and physicians to distinguish between these two diagnoses clinically. Since ATD-induced neutropenia is associated with increased risk of infections, it is plausible that this rare adverse effect of antithyroid drugs favours progression of COVID-19, possibly through a reduction in the innate immune response or through generalised immune suppression. Importantly, 50% of patients who died from COVID-19 disease had co-existent secondary bacterial infections, and the presence of neutropenia is highly likely to increase the risk of such co-infections and hence affect severity of disease (12).
- At present, most countries do not recommend nasopharyngeal swabs in patients with only mild flu-like symptoms. We recommend considering the discontinuation of ATD in patients with symptoms suggestive of neutropenia. An urgent full blood count (FBC) to measure neutophil count should be strongly advised (8). Depending upon the manifestations and history, testing for COVID-19 should also be considered. As per standard practice, we recommend all patients starting ATDs be given written information with instructions on what to do if they develop symptoms suggestive of neutropenia. Of note, lymphopenia and thrombocytopenia are seen in COVID-19 infection (13), but these do not represent indications to stop antithyroid drugs.
- *Management of neutropenia in a resource-limited setting*: It is likely that healthcare resources will be severely limited over the coming weeks/months. It may not be always possible to check a full blood count at the onset of symptoms suggestive of neutropenia; in this extraordinary situation, we suggest that patients stop the ATD and restart 1 week later if symptoms have resolved. A reduction in the dose of antithyroid drugs or use of an alternative drug may be considered. If symptoms worsen during the period off ATDs or recur after recommencing the drug, the patient should seek urgent medical attention; in such situations, performing a neutrophil count is essential and alternative treatments for hyperthyroidism should be considered.
- *Surgical treatment of hyperthyroidism*: Elective surgical procedures have been cancelled in most countries, so it is unlikely that those awaiting thyroidectomy for benign disease will have thyroid surgery during the outbreak. On a case-by-case basis, uncontrolled thyrotoxicosis in patients who have developed significant adverse effects from antithyroid drugs may require urgent thyroid surgery (14).
- *Radioiodine administration for hyperthyroidism*: Elective radioiodine administration with 131-I has been postponed in most countries both for benign and for malignant thyroid diseases. This is based on prioritisation of delivery of emergency care with re-location of healthcare staff and resources as well as anticipated difficulties with patients being unable to adhere to radiation protection guidance during the COVID-19 pandemic. We feel confident that, in most cases, there will be no long-term adverse effects from postponing 131-I therapy. Importantly, it is crucial to identify those patients who have undergone 131-I treatment for hyperthyroidism in the months before the COVID pandemic and to adopt a low threshold for commencing levothyroxine therapy if hypothyroid symptoms develop. In patients with Graves’ disease, when 131-I treatment is urgently required, prophylactic steroid treatment should be given when indicated (15).

- *Diagnosing hyperthyroidism*: Where possible, the diagnosis of hyperthyroidism should continue to be based on clinical suspicion, supported by characteristic biochemistry results. If facilities are available, it remains important to check TSH-receptor antibodies (TRAb) to identify the underlying diagnosis (8, 9). This is particularly important as most centres have suspended the use of diagnostic isotope scanning during the COVID-19 crisis in view of staff and resource unavailability and in order to reduce footfall in hospitals. Thyroid ultrasonography may aid in defining the underlying aetiology (8), although with a reduction in the availability of hospital appointments, and since treatment options for hyperthyroidism are limited regardless of the underlying aetiology, the role of this imaging modality is likely to be limited. Determination of malignancy risk associated with palpable thyroid nodules can usually be postponed, unless particular characteristics (rapid growth, highly suspicious features at palpation or imaging) indicate a high-risk thyroid cancer.
- *Regimens for treatment of thyrotoxicosis*: Patients whose hyperthyroidism is well controlled with a titration or block-and-replace regimen (BRR) should continue their treatment without changes. However, over the coming weeks to months, since it may become difficult to undertake biochemical monitoring of thyrotoxicosis, BRRs should be considered as initial treatment, especially in patients presenting with new or relapsed hyperthyroidism. BRRs generally have similar efficacy and long-term cure rates as titration regimens but usually reduce the amount of thyroid function testing that is required and allow euthyroidism to be achieved and maintained in the majority of patients with hyperthyroidism, irrespective of aetiology (9, 10). Suggested BBRs for adults and children are outlined in Figs 1 and 2, respectively. Resumption of thyroid function testing should be undertaken as soon as is practicable. If patients develop significant symptoms, while being treated according to the algorithm, thyroid function should be tested and the patient discussed with an endocrinologist who can advise on further management. Assessment of thyroid status is particularly important if new or worsening features of thyroid eye disease are present.

### Hypothyroidism

- *Management of hypothyroidism*: No particular changes are envisaged relating to the diagnosis and treatment of hypothyroidism during the COVID-19 crisis (16). Patients are advised to continue the same form and dosage of thyroid hormone replacement therapy. Regular blood test monitoring may be difficult, but when patients on thyroid hormone replacement feel significantly unwell or if there are significant weight changes, thyroid function testing, preferably with measurement of serum TSH and fT4, is recommended in order to adjust medication if needed.
- *Neonatal Screening for congenital hypothyroidism*: It is crucial that screening is maintained with particular attention to avoid delayed diagnosis and treatment. Careful and regular monitoring of thyroid function should be continued to optimise thyroid function, particularly during the first months of life (17). It may be necessary for highly specialized screening laboratories to cooperate or for independent facilities to be built in order ensure appropriate quarantining and infection control measurements. In addition, appropriate measures to ensure timely transport and processing of specimens, for example, through use of courier services are needed.
- *Supply of thyroid hormone replacement*: During the COVID-19 pandemic, stockpiling of any medication should be avoided, in order to ensure sufficient supply for all in the community. We recommend that patients ensure they have adequate supply of medication.

## Which patient groups are at particular risk from COVID-19 infection?

- *Patients with thyroid eye disease on immuno* *-suppressive medication*: Patients with thyroid eye disease who are undergoing treatment with immunosuppressive agents are considered to be extremely vulnerable and at very high risk of severe illness from coronavirus (COVID-19) and should be advised to self-isolate for at least 12 weeks (18). This includes patients on glucocorticoids at immunosuppressive doses as well as those on other immunosuppressive agents such as mycophenolate, azathioprine and biological agents including teprotumumab, rituximab and tocilizumab (19). It is particularly important to reduce the risk of progression of ophthalmopathy during this pandemic: advice including the discontinuation of cigarette smoking and/or selenium supplementation should be reinforced (15). In addition, conjunctivitis has been reported to be a manifestation of COVID-19 and SARS-CoV-2 mRNA has been detected in tear drops (20). Patients with ophthalmopathy who develop COVID-19 may therefore represent a significant risk of infection, especially if they have prominent ocular soft tissue involvement. Patients with thyroid eye disease should be advised to avoid tear drop leakage and careful adherence to personal protective equipment guidance is advised for healthcare professionals who are involved in their management.
- *Pregnant women with hypo- or hyperthyroidism*: Pregnant women are advised to be particularly stringent about adhering to social distancing measures as they are at increased risk of developing more severe COVID-19 disease (21). General advice regarding the management of the fetus and newborn during the COVID-19 pandemic (22) is outside the scope of this manuscript. Expecting mothers with treated hypothyroidism should continue to follow the advice to increase the dose of levothyroxine as soon as they have a positive pregnancy test, for example, by doubling the current dose of levothyroxine on 2 days of the week. Ideally, thyroid function should be checked using population and trimester-specific reference ranges on a regular basis (23, 24), although in women whose thyroid function is stably controlled, the frequency of testing may be reduced, depending on available infrastructure, in order to reduce footfall in blood testing facilities. The usual principles of treatment with ATD, preferably with propylthiouracil before and in the first trimester of pregnancy, should be adhered to and the lowest possible dose of ATDs should be used (23). Measurement of TRAb and regular thyroid function testing is advised in those treated with ATDs, available resources and infrastructure allowing.

## How should endocrine services for thyroid dysfunction be remodelled in the acute crisis?

- *Telephone and video consultations*: In liaison with primary-care physicians, endocrinologists can provide support in the form of advice and guidance letters, telephone advice, virtual outpatient clinics or face-to-face appointments, as dictated by the clinical urgency and availability of staff resources. If there are clinical concerns during the virtual appointment, it is appropriate to arrange face-to-face consultations in selected patient groups. Many centres have set up dedicated helplines maintained by dedicated endocrine-specialist nurses supported by endocrinologists.
- *Remote monitoring services*: Some centres already have established remote follow-up services for patients on a prolonged course or long-term antithyroid drugs, for those on thyroid hormone replacement for endogenous or iatrogenic hypothyroidism and for close follow-up of patients who have undergone radioactive iodine treatment. These services should continue if staff are available to perform them.
- *Face-to-face appointments*: Patients with new-onset or worsening thyroid eye disease, those with enlarging goitres causing symptoms of obstruction and patients who are not responding to standard treatment measures as expected may require appointments in person for a thorough clinical assessment. We anticipate that this is only required in a minority of patients.
- *Satellite blood-testing services*: The management of thyroid dysfunction is heavily reliant on biochemical testing. Many centres have established peripheral ‘pods’ for blood taking, and tests required for managing thyroid dysfunction do not require specific handling, making such facilities ideal for these particular patients.

## What might be the longer-term consequences for service provision?

- Patients with thyroid dysfunction will not be managed as closely as would be ideal in non-crisis situations. Patients with severe hyper-and hypothyroidism should be prioritised. The underlying aetiology of those with hyperthyroidism will not be identified in a significant number of patients and those with toxic nodular hyperthyroidism will not receive the most appropriate treatment, namely 131-I treatment or thyroid surgery. In addition, there will be a significant back-log of patients who have not received timely appointments and who will need to be reviewed in person or remotely depending on the duration of the crisis and the clinical needs.
- It seems plausible that the experience with remote and virtual follow-up of patients will translate into a number of systems that can be continued and maintained following the COVID-19 pandemic, resulting in more efficient management of patients with thyroid dysfunction.

## Which online resources are available?

Additional information is available on government, professional societies and patient-support-group websites. These include:

- The latest NHS advice to patients: https://www.nhs.uk/conditions/coronavirus-covid-19/#
- Social distancing advice from the UK government: https://www.gov.uk/government/publications/covid-19-guidance-on-social-distancing-and-for-vulnerable-people/guidance-on-social-distancing-for-everyone-in-the-uk-and-protecting-older-people-and-vulnerable-adults
- BTF information for patients: https://www.btf-thyroid.org/news/thyroid-disease-and-coronavirus-covid-19
- Information for clinicians: https://www.england.nhs.uk/coronavirus/
- SfE resources page: https://www.endocrinology.org/clinical-practice/covid-19-resources-for-managing-endocrine-conditions/?utm_campaign=298983_Covid-19%20resources&utm_medium=email&utm_source=SfE&dm_i=52U8,6EP3,9STD7,O1VU,1
- Information for caregivers and patients: https://endo-ern.eu/covid-19/covid-19-european-initiatives/#europeanmedicinesagencyema
- Information from Italian National Health System: https://www.epicentro.iss.it/coronavirus/
- Information from ETA: https://www.eurothyroid.com/news/covid-19-thyroid-diseases.html
- Information from ATA: https://www.thyroid.org/covid-19/

## Disclaimer

Due to the emerging nature of the COVID-19 crisis, this document is not based on extensive systematic review or meta-analysis, but on rapid expert consensus. The document should be considered as guidance only; it is not intended to determine an absolute standard of medical care. Healthcare staff need to consider individual circumstances when devising the management plan for a specific patient.

## Declaration of interest

Juliane Léger is Deputy Editor of the *European Journal of Endocrinology*. She was not involved in the editorial or peer-review process of this paper, on which she is listed as an author. Edward Visser is Associate Editor for the *European Journal of Endocrinology*. He was not involved in the editorial or peer-review process of this paper, on which he is listed as an author. The other authors have nothing to disclose.

## Funding

This guidance did not receive any specific grant from any funding agency in the public, commercial or not-for-profit sector.
